# Optimizing Energy Structure in Low-Protein Diets Reduced Body Fat Deposition in Geese

**DOI:** 10.3390/vetsci13060504

**Published:** 2026-05-22

**Authors:** Xucheng Zheng, Jie Shen, Zhi Yang, Wei Wang, Xuan Li, Haiming Yang, Zhiyue Wang

**Affiliations:** 1 College of Animal Science and Technology, Yangzhou University, Yangzhou 225009, China; dx120230175@stu.yzu.edu.cn (X.Z.); sj990018@163.com (J.S.); tzznwangwei@sina.com (W.W.); liooo1111@163.com (X.L.); hmyang@yzu.edu.cn (H.Y.); 2 Institutes of Agricultural Science and Technology Development (Joint International Research Laboratory of Agriculture & Agri-Product Safety), Yangzhou University, Yangzhou 225009, China; zhiyang@yzu.edu.cn

**Keywords:** AMPK, energy supply, fatty acid, geese, low-protein diet

## Abstract

This study examined the effects of dietary crude protein (CP) level and starch: fat ratio (SFR) on glucose and lipid metabolism in geese. A total of 360 male Jiangnan White geese were allocated to a 3 × 2 factorial arrangement with two CP levels (14.5% and 16.5%) and three SFRs (SFR_20:1_, SFR_11:1_, and SFR_5:1_) from 28 to 63 days of age. Under the low-protein condition, Both the SFR_11:1_ and SFR_5:1_ group enhanced body weight of geese at 63 d, but SFR _5:1_ increased subcutaneous and abdominal fat deposition. Dietary SFR changed liver cholesterol metabolism and glycogen content, while CP levels mainly affected the activity of enzymes related to liver glucose and lipid metabolism: 14.5% CP increased AMPK and ACC activity, but decreased FAS, CS and G6PC activity. Both CP level and SFR altered muscle fatty acid composition, but the effect of SFR was usually more significant. An SFR of 11:1 was beneficial for improving the muscle fatty acid profile. Gene expression analysis further revealed that low protein compensatorily regulated liver energy metabolism, while excessive fat in low SFR diets led to lipid metabolism disorders. In conclusion, optimizing the energy structure of low-protein diets, especially by maintaining a medium SFR (11:1), could improve glucose and lipid metabolism in geese while increasing body weight.

## Simple Summary

Low-protein diets are widely used in goose production, but they could also lead to excessive fat deposition due to more starch content and energy imbalance. This study aimed to investigate whether adjusting the starch: fat ratio in the low-protein diet could maintain normal glucose and lipid metabolism in geese. A total of 360 male geese received diets containing two crude protein levels (14.5% and 16.5%) and three SFRs (SFR_20:1_, SFR_11:1_, and SFR_5:1_). A lower SFR increased body fat deposition, Liver cholesterol accumulation, and reduced liver glycogen stores. Dietary protein level mainly influenced Liver AMPK-related enzyme activities, whereas SFR exerted a stronger effect on Liver glucose and lipid metabolism as well as muscle fatty acid composition. In particular, an SFR of 11:1 better maintained the unsaturated fatty acid profile of breast muscle than an SFR of 5:1. Overall, improving the energy structure of low-protein diets, especially by avoiding an excessively low SFR, might benefit glucose and lipid metabolism and help maintain meat lipid quality in geese.

## 1. Introduction

The main energy source for poultry diets is grains (starch). Poultry can usually digest starch efficiently, with the jejunum being the main site of starch digestion [1,2]. However, the rate and extent of starch digestion are affected by factors such as starch structure, the encapsulation effect of protein matrix, feed viscosity, and feed processing conditions [3]. In contrast, lipid digestion and absorption are more complex because they depend on the emulsification of bile acids, the hydrolysis of pancreatic lipases, and the formation of mixed micelles [4]. Starch and fat together determine the energy supply pattern of poultry diets: starch mainly determines glucose utilization, while fat plays an important role in dietary energy density and digestive environment. Therefore, the dietary starch: fat ratio (SFR) may affect the digestibility of nutrients, energy utilization efficiency, and carcass fat deposition [5].

Starch and fat provide energy in very different ways. First, their energy release kinetics are different. In birds, starch is mainly hydrolyzed in the small intestine by pancreatic α-amylase and brush border enzymes, and is eventually absorbed by the intestine as glucose through glycosidic bond cleavage [6]. However, fat exists mainly as triglycerides, which must first be emulsified by bile salts, and then hydrolyzed by pancreatic lipases into monoglycerides and free fatty acids, which are then absorbed in mixed micelles [7]. Therefore, the digestion and absorption of lipids is more complex than that of starch. Second, the absorption rates of these two nutrients are also different. Generally, starch-derived glucose is absorbed faster because it can pass directly across intestinal epithelial cells via glucose transporters [8]. In contrast, fatty acids must first be absorbed into micelles, and long-chain fatty acids must be further re-esterified into triglycerides and packaged into chylomicrons for transport, which slows down the entire process.

Energy supply processes require coordination of glucose and lipid metabolism, and AMP-activated protein kinase (AMPK) is a classic and key signaling pathway in this process. Once activated, AMPK can inhibit the de novo synthesis of fatty acids by inhibiting the activity of acetyl-CoA carboxylase (ACC) [9]. AMPK can also limit glycogen accumulation by inhibiting the phosphorylation of glycogen synthase (GS) [10] and reduce the biosynthesis of hexosamine by phosphorylating glutamine-6-phosphate fructose transaminase 1 (GFPT1) [11].

Previous studies have shown that low-protein diets can reduce nitrogen excretion without impairing goose growth performance [12,13]. In China, low-protein feeding has gradually become common in goose production, and dietary protein during the finishing period is sometimes reduced to 12% or even lower. Although this practice can increase body weight at market age (63 or 70 days), it may also promote abdominal fat deposition because the dietary starch: protein ratio rises markedly [14]. Moreover, the use of large amounts of oil (more than 5%) during fattening can seriously disturb lipid metabolism and ultimately impair meat quality [15]. Improving nutrient partitioning efficiency has therefore become an important issue in goose production.

Therefore, it is hypothesized that a moderately low starch-to-fat ratio (SFR) would better maintain glucose and lipid homeostasis and improve meat fatty acid profile in geese fed low-protein diets, whereas an excessively low SFR would lead to lipid metabolism disorders and excessive fat deposition. Therefore, the significance of this study is to identify an optimal SFR in low-protein diets that can balance growth performance and metabolic health, providing a practical reference for formulating low-protein, energy-optimized diets for geese.

## 2. Materials and Methods

### 2.1. Experimental Diets and Design, Bird Management

A total of 360 healthy commercial male Jiangnan White geese (medium-sized white goose synthetic line, raised for meat production and usually marketed at 63 days of age) with similar body weight at 28 days of age were obtained from Changzhou Siji Poultry Co., Ltd. (Changzhou, China). Birds were randomly assigned to six treatment groups with six replicates per treatment and 10 goslings per replicate. The experiment followed a 3 × 2 factorial design with two crude protein levels (16.5% and 14.5%) and three starch-to-fat ratios (20:1, 11:1, and 5:1).

Soybean oil was used to replace part of the corn in order to adjust the dietary SFR. The two crude protein levels were chosen based on the nutritional requirements of finishing geese: 16.5% CP represents a standard or near-recommended level, while 14.5% CP is a moderately reduced level (2 percentage points lower) that is commonly used in low-protein diet studies in poultry and has been shown to affect nitrogen excretion without severely compromising growth performance [12]. The three SFRs were designed to cover a range from high starch to high fat: 20:1 mimics a conventional corn-soybean meal diet with minimal added fat; 11:1 represents a moderate SFR achieved by adding approximately 3–4% oil, which is a common adjustment in commercial goose diets; and 5:1 represents a very low SFR (high fat) with about 5–6% oil, which is occasionally used in fattening practices but may induce metabolic disorders. This gradient allows to systematically evaluate the dose-dependent effects of SFR on glucose and lipid metabolism and to identify an optimal SFR (e.g., moderate) for low-protein diets. All diets were formulated to the same apparent metabolizable energy level. The feed formulations and nutrient levels for different treatments are shown in Table 1.

Throughout the experimental period, geese were housed in floor pens with plastic-wire flooring, and each pen measured 1.9 m × 1.5 m (2.85 m^2^). Experimental diets were provided in mash form, and birds had free access to feed and water. Natural light was used during the whole trial. The rearing period lasted from 28 to 63 days of age. During the experiment, the house was kept clean and well ventilated, and the ambient temperature was maintained at approximately 25 °C.

### 2.2. Sample Collection, Chemical Analyses and Calculations

#### 2.2.1. Body Fat Composition

All experimental geese were weighed after fasting for 6 h, then stunned with electric shock (65 V, 86 mA, 400 Hz for 18 s per bird) and euthanized by bleeding. Body fat traits were determined according to Performance Terminology and Measurements for Poultry (NY/T 823-2020) [16]. The equations used for calculating the different indices were as follows:

Skin and subcutaneous fat thickness: a cross-shaped incision was made in the skin to expose the tailbone, and measurements were taken 1 cm above the tailbone with calipers three times; the average value was used.

Skin and subcutaneous fat yield = (skin and subcutaneous fat weight + abdominal fat weight)/ (eviscerated weight + abdominal fat weight) × 100%;

Abdominal fat yield = abdominal fat weight/(abdominal fat weight + eviscerated weight) × 100%;

Intestinal fat yield = intestinal fat weight/eviscerated weight × 100%;

Leg fat yield = fat around the legs/eviscerated weight × 100%;

Body fat yield = (skin and subcutaneous fat weight + abdominal fat weight + intestinal fat weight  + leg fat weight)/eviscerated weight × 100%.

#### 2.2.2. Serum and Liver Lipid Metabolites

Before slaughter, 5 mL of blood was collected from the brachial vein of one goose per replicate with body weight close to the replicate mean. Serum was separated by centrifugation at 3500 rpm for 10 min using a Cence DL-5M low-speed refrigerated centrifuge (Hunan Xiangyi Laboratory Instrument Development Co., Ltd, Changsha, China) and stored at −20 °C until analysis. After the geese were slaughtered, samples from the right side of the liver were rapidly collected and stored at −80 °C. Serum and Liver triglyceride (TG), total cholesterol (TCHO), high-density lipoprotein cholesterol (HDL-c), and low-density lipoprotein cholesterol (LDL-c) were measured using commercial kits supplied by Nanjing Jiancheng Biotechnology Institute (Nanjing, China). The catalog numbers were as follows: TG assay kit (A110-1-1); TCHO assay kit (A111-1-1); HDL-c assay kit (A112-1-1); and LDL-c assay kit (A113-1-1).

#### 2.2.3. Liver and Muscle Glycogen Content

After slaughter, samples of liver (right side), breast muscle, and leg muscle were collected immediately and stored at −80 °C for subsequent analysis. Glycogen content in liver and muscle was determined using glycogen assay kits (A043-1-1) provided by Nanjing Jiancheng Bioengineering Institute (Nanjing, China).

#### 2.2.4. Enzymes Activity Related to Liver Glucose and Lipid Metabolism

The activities of citrate synthase (CS), glucose-6-phosphatase (G6PC) and AMP-activated protein kinase (AMPK) in liver tissue were determined using ELISA kits (ml092812, ml037561 and ml060852, respectively) from Shanghai Enzyme-linked Biotechnology Co., Ltd. (Shanghai, China). The activities of fatty acid synthase (FAS) and acetyl-CoA carboxylase (ACC) were determined using ELISA kits (YB-FAS-1 and YB-ACC-1, respectively) and purchased from Shanghai Yubo Biotechnology Co., Ltd. (Shanghai, China).

#### 2.2.5. Muscle Amino Acid and Fatty Acid Profile

Muscle amino acid content was determined according to Chinese National Standard (GB 5009.124-2016) [17]. Briefly, approximately 1 g of muscle sample was hydrolyzed with 6 mol/L HCl at 110 °C for 24 h to release free amino acids. After filtration and purification using a C18 solid-phase extraction column, the hydrolysate was analyzed using an automatic amino acid analyzer equipped with an ion-exchange chromatographic column and post-column ninhydrin derivatization. The separated amino acids were detected spectrophotometrically at 570 nm and 440 nm (for proline), and quantified by external standard method. A total of 16 amino acids were determined, including aspartic acid, threonine, serine, glutamic acid, proline, glycine, alanine, valine, methionine, isoleucine, leucine, tyrosine, phenylalanine, histidine, lysine, and arginine.

Muscle fatty acid content was determined according to Chinese National Standard (GB 5009.168-2016) [18]. Total lipids were extracted from muscle samples, and fatty acids were converted to fatty acid methyl esters (FAMEs) via transesterification. The FAMEs were then separated and quantified using a gas chromatograph equipped with a flame ionization detector (GC-FID). Separation was achieved on a capillary column (e.g., 100 m × 0.25 mm × 0.2 μm or similar), with hydrogen or helium as the carrier gas, and an appropriate temperature gradient program. Fatty acids were identified by comparison with retention times of a 36-component fatty acid methyl ester standard mixture, and quantified by area normalization.

#### 2.2.6. Relative Expression Level of Gene mRNA

Total RNA was isolated from liver tissue using a Total RNA Extraction Kit (Shanghai Yeasen Biotechnology Co., Ltd., Shanghai, China). After RNA concentration was measured, reverse transcription was carried out with a reverse transcription kit (Shanghai Yeasen Biotechnology Co., Ltd., Shanghai, China) following the manufacturer’s instructions. The resulting cDNA was diluted (1:5) and used for quantitative real-time PCR analysis. β-actin served as the internal reference gene. Each 20 μL reaction mixture contained 10 μL Hieff^®^ qPCR SYBR Green Master Mix (No Rox) (Cat. No. 11201ES03, Yeasen Biotechnology, Shanghai, China), 0.4 μL each of forward and reverse primers (10 μM), 2 μL of cDNA template, and 7.2 μL RNase-free water. The PCR program was performed on a CFX96 Touch Real-Time PCR Detection System (Bio-Rad, Hercules, CA, USA) controlled by CFX Manager Software version 3.1, and consisted of an initial denaturation at 95 °C for 5 min followed by 40 cycles of 95 °C for 10 s and 60 °C for 30 s. A melting curve analysis was performed after each run to confirm amplification specificity. The raw Ct values of the target genes were normalized to the Ct values of β-actin to obtain ΔCt (ΔCt = Ct_target–Ct_β-actin). The ΔCt values for each target gene were then calibrated against the ΔCt of the control group (LPSFR20:1, 14.5% CP + SFR 20:1) to generate ΔΔCt (ΔΔCt = ΔCt_treatment–ΔCt_calibrator). The control group was assigned a relative expression value of 1 by definition. The relative expression level of each target gene was finally calculated as 2^^ΔΔCt^. Primers for PPARα, CPT-1, CS, SREBP-1, ACC, FAS, and β-actin were synthesized by Genewiz Biotechnology Co., Ltd. (Suzhou, China). Primer information for genes related to glucose and lipid metabolism is listed in Table 2.

### 2.3. Statistical Analysis

Statistical analysis were performed using SPSS 26.0 (SPSS Inc., Chicago, IL, USA). Data were analyzed by two-way ANOVA according to a 3 × 2 factorial arrangement using the General Linear Model (GLM) procedure. The model included the main effects of CP and AM/AP ratio, as well as their interaction. Prior to analysis, data were checked for normality and homogeneity of variance. When a significant interaction was detected, simple effects were further analyzed. When no significant interaction was observed, the main effects were interpreted. Multiple comparisons among treatment means were performed using Fisher’s least significant difference (LSD) test when appropriate. Results are presented as means ± standard error of the mean. Statistical significance was declared at *p* < 0.05, and 0.05 ≤ *p* < 0.10 was considered a tendency.

## 3. Results

### 3.1. Body Fat Composition

Table 3 shows the body fat traits of geese at 63 days of age under the 14.5% dietary protein level. LPSFR_20:1_ group had lower live body weight compared to the other two groups (*p* < 0.05). As dietary SFR decreased, skin and subcutaneous fat thickness, skin and subcutaneous fat yield, abdominal fat yield, and body fat yield all increased (*p* < 0.05). By contrast, intestinal fat yield and leg fat yield were not affected by dietary SFR (*p* > 0.05).

### 3.2. Serum and Liver Lipid Metabolites

As presented in Table 4, dietary CP level did not influence serum or Liver lipid-related indices (*p* > 0.05). Variations in dietary SFR did not alter serum TCHO or TG concentrations in 63-day-old geese (*p* > 0.05), but did affect HDL-c and LDL-c levels (*p* < 0.05). Serum HDL-c in the SFR5:1 group was higher than that in the SFR_20:1_ and SFR11:1 groups (*p* < 0.05), whereas serum LDL-c concentrations in the SFR_5:1_ and SFR_11:1_ group were lower than that in the SFR_20:1_ group (*p* < 0.05). Dietary SFR did not affect Liver TG or HDL-c concentrations (*p* > 0.05), but influenced Liver TCHO and LDL-c (*p* < 0.05). Specifically, Liver TCHO contents in the SFR_11:1_ and SFR_20:1_ group were lower than those in the SFR_5:1_ group (*p* < 0.05). Likewise, Liver LDL-c content was higher in the SFR_5:1_ group than in the other two SFR groups (*p* < 0.05). A significant CP × SFR interaction was detected for serum HDL-c (*p* = 0.021). In addition, interaction effects tended to occur for Liver TCHO (*p* = 0.060) and Liver LDL-c (*p* = 0.065).

### 3.3. Liver and Muscle Glycogen Content

Results for liver and muscle glycogen are shown in Table 5. Dietary SFR significantly affected Liver glycogen content (*p* < 0.05), and the SFR_5:1_ group showed lower liver glycogen than the SFR_20:1_ and SFR_11:1_ group. In contrast, SFR did not influence glycogen content in breast or leg muscle (*p* > 0.05). Altering dietary CP level did not significantly affect glycogen content in liver or muscle (*p* > 0.05).

### 3.4. Enzymes Activity Related to Liver Glucose and Lipid Metabolism

As shown in Table 6, dietary SFR did not affect the Liver activities of FAS, AMPK, ACC, CS, or G6PC (*p* > 0.05). In contrast, these enzymes responded to CP level. Compared with the 16.5% CP diet, the 14.5% CP diet increased Liver AMPK and ACC activities (*p* < 0.05), whereas it decreased the activities of FAS, CS, and G6PC (*p* < 0.05).

### 3.5. Amino Acid Profile in Muscles

The effects of dietary treatments on muscle amino acid composition are presented in Table 7 and Table 8. In breast muscle, the Pro content of the SFR_5:1_ group was lower than that of the other SFR groups (*p* < 0.05). In leg muscle, Pro content in the SFR_20:1_ group was lower than that in the other SFR groups (*p* < 0.05). In addition, the 14.5% CP diet increased Ser content in leg muscle compared with the 16.5% CP diet (*p* < 0.05).

### 3.6. Fatty Acid Profile in Muscles

Table 9 and Table 10 show that both dietary CP level and SFR influenced the fatty acid composition of breast and leg muscles in geese at 63 d, although the response patterns differed between the two muscles. In breast muscle, relative to the 16.5% CP diet, the 14.5% CP diet increased the contents of C14:0, C16:0, C18:0, C20:0, C18:2 n-6, C18:3 n-3, total saturated fatty acids (SFA), total polyunsaturated fatty acids (PUFA), n-6 PUFA, n-3 PUFA, and total fatty acids, while reducing the proportion of C22:1 n-9 (*p* < 0.05). Changes in dietary SFR also altered breast muscle fatty acid composition. The SFR_11:1_ diet increased C18:1 n-9, total monounsaturated fatty acids (MUFA), while it decreased C20:0, compared with the SFR20:1 and SFR5:1 group (*p* < 0.05). The SFR5:1 diet increased C14:0, C16:0, and C18:0 contents relative to the other SFR treatments (*p* < 0.05).

In leg muscle, compared with the 16.5% CP diet, the 14.5% CP diet increased the contents of C4:0, C20:2, C20:3 n-3, and C20:4 n-6 (*p* < 0.05), whereas the 16.5% CP diet increased C22:1 n-9 (*p* < 0.05). Dietary SFR also modified several leg muscle fatty acid indices. The SFR_11:1_ diet increased C20:1, C22:1 n-9, and MUFA compared with the other SFR groups, whereas the SFR_20:1_ diet increased C20:5 n-3, n-3 PUFA, and the n-3: n-6 ratio (*p* < 0.05). In addition, the 14.5% CP level increased C16:0 and SFA under the SFR5:1 condition, indicating a significant CP × SFR interaction for these traits (*p* < 0.05).

### 3.7. Relative Expression of Genes Related to Glucose and Lipid Metabolism

**Figure 1:**
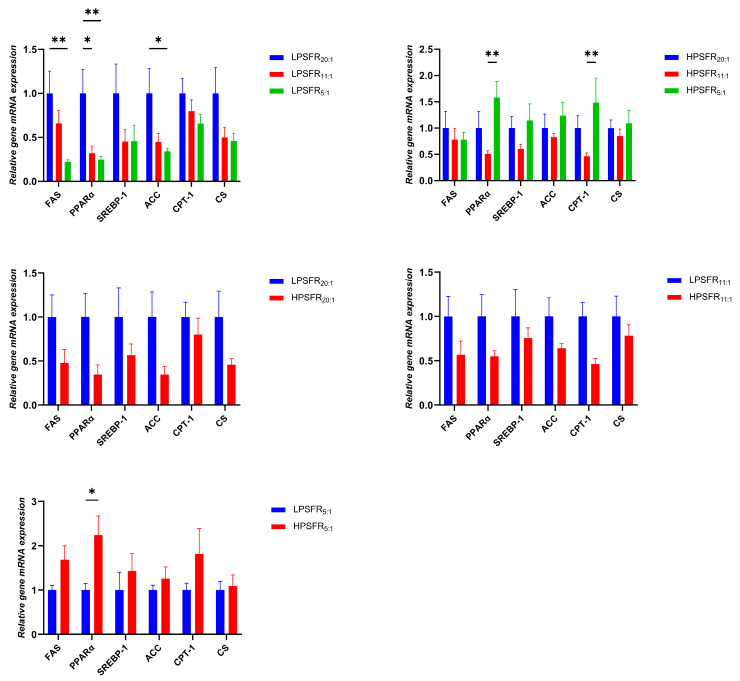
Effects of different dietary treatments (main effects) on the expression levels of genes related to glucose and lipid metabolism in geese at 63 d. LPSFR20:1 (CP, 14.5% + SFR, 20:1), HPSFR20:1 (CP, 16.5% + SFR, 20:1), LPSFR11:1 (CP, 14.5% + SFR, 11:1), HPSFR11:1 (CP, 16.5% + SFR, 11:1), LPSFR5:1 (CP, 14.5% + SFR, 5:1), HPSFR5:1 (CP, 16.5% + SFR, 5:1). FAS, fatty acid synthase; PPARα, peroxisome proliferator-activated receptor alpha; SREBP-1, sterol regulatory element-binding protein 1; ACC, acetyl-CoA carboxylase; CPT-1, carnitine palmitoyltransferase 1; CS, citrate synthase. Asterisks indicate significant differences between groups (* *p* < 0.05, ** *p* < 0.01).

**Figure 2:**
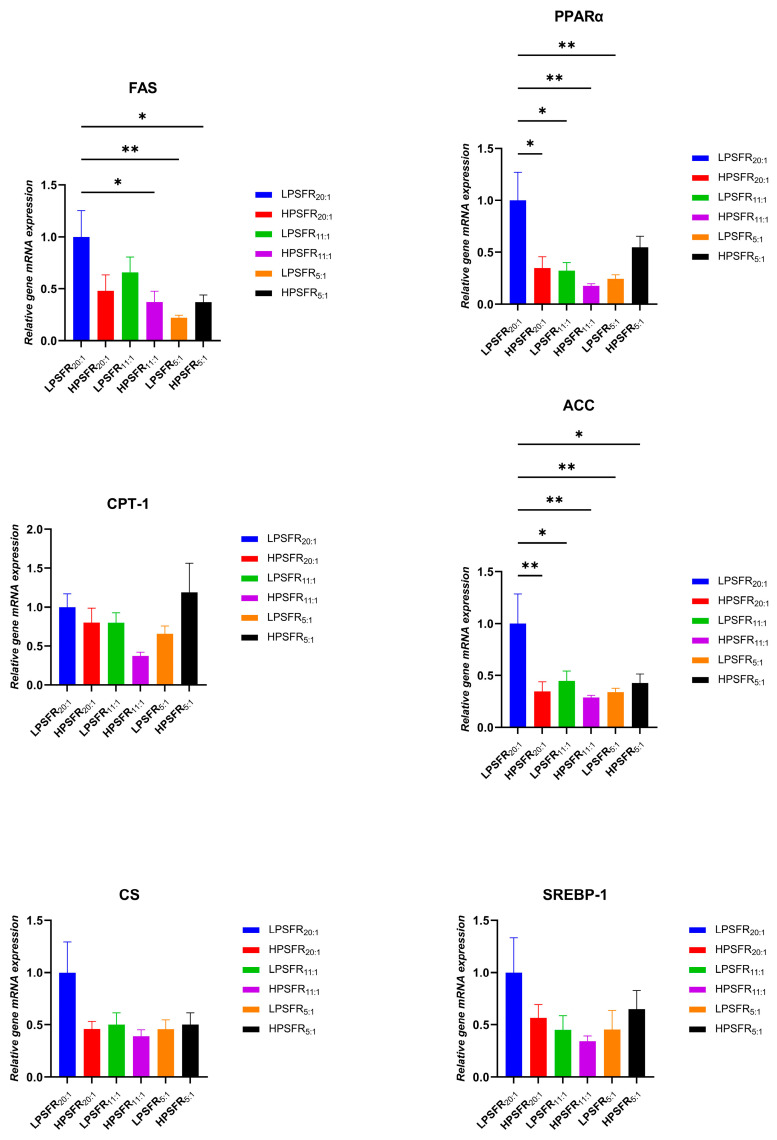
Effects of different dietary treatments (interactions) on the expression levels of genes related to glucose and lipid metabolism in geese at 63 d. LPSFR_20:1_ (CP, 14.5% + SFR, 20:1), HPSFR_20:1_ (CP, 16.5% + SFR, 20:1), LPSFR_11:1_ (CP, 14.5% + SFR, 11:1), HPSFR_11:1_ (CP, 16.5% + SFR, 11:1), LPSFR_5:1_ (CP, 14.5% + SFR, 5:1), HPSFR_5:1_ (CP, 16.5% + SFR, 5:1). FAS, fatty acid synthase; PPARα, peroxisome proliferator-activated receptor alpha; SREBP-1, sterol regulatory element-binding protein 1; ACC, acetyl-CoA carboxylase; CPT-1, carnitine palmitoyltransferase 1; CS, citrate synthase. Asterisks indicate significant differences between groups (* *p* < 0.05, ** *p* < 0.01).

The effects of different dietary treatments on the mRNA expression of lipid metabolism-related genes are shown in Figure 1 and Figure 2. Under the low-protein condition, as SFR decreased, the relative expression of FAS, PPARα, and CPT-1 declined significantly (*p* < 0.05). Under the high-protein condition, Compared with the SFR11:1 group, the relative mRNA expression levels of PPARα and CPT-1 were significantly upregulated in the SFR5:1 group (*p* < 0.05). Further comparisons between CP levels within each SFR showed that, only under the SFR5:1 diets, the low-protein diet decreased the expression of PPARα (*p* < 0.05). The interaction effect showed that the relative mRNA expression levels of FAS, PPARα, and ACC in the LPSFR20:1 group were higher than those in the other groups (*p* < 0.05).

## 4. Discussion

In the present experiment, serum lipid metabolites remained comparatively stable across dietary treatments, suggesting that systemic lipid metabolism was largely maintained within the tested nutrient range. In contrast, liver cholesterol-related traits were more responsive. The reduction in dietary protein was mainly reflected in a decline in HDL-c, which may be associated with changes in liver apolipoprotein synthesis, cholesterol transport, or lipoprotein turnover [19]. Meanwhile, geese fed an SFR_5:1_ diet showed higher concentrations of TCHO and LDL-c in birds’ livers, indicating that increased dietary fat promotes cholesterol accumulation in the liver. This was consistent with previous findings [20,21]. Notably, liver TG levels remained stable, suggesting that changes in SFR in this experiment had a greater impact on cholesterol metabolism than triglyceride metabolism. The significant CP × SFR interaction of serum HDL-c and the trend of interaction between liver TCHO and LDL-c suggested that the metabolic component of liver lipid metabolism in relation to dietary energy structure depends on protein supply, which may be achieved by altering liver lipid transport and nutrient distribution.

The experiment also showed that dietary energy structure had a greater impact on liver glycogen than CP level; similar results were found in the experiment by Xu et al. [22]. As the starch-to-fat ratio (SFR) decreased from 20:1 to 5:1, liver glycogen content decreased, indicating that reduced dietary starch supply weakens liver glycogen synthesis. Changes in SFR may affected glycogen reserves primarily through liver glucose flux and hormonal regulation [23]. In contrast, glycogen concentrations in breast and leg muscles did not change significantly, possibly because muscles maintained relatively stable levels through glycogen turnover control. Since the liver was the core organ for short-term glucose regulation, it is expected to respond more readily to changes in carbohydrate supply than skeletal muscle [24].

The overall amino acid composition of goose muscle showed limited sensitivity to different dietary treatments. In leg muscle, only Ser content changed with variations in dietary CP levels. This difference may reflect subtle changes in nutrient distribution under low protein supply, as Ser is involved in intermediate metabolism, immune regulation, lipid metabolism, and protein synthesis [25,26]. Furthermore, a decrease in SFR reduced Ile content in pectoral muscle, possibly because when a large amount of fat supplied energy, the body may activate branched-chain amino acid transaminases, leading to the extensive oxidation and decomposition of ile in muscle, thus reducing its deposition in muscle [27]. As for the changes in Pro, this might be because Pro, as an important glucogenic amino acid, entered the tricarboxylic acid cycle and participated in gluconeogenesis.

Both CP levels and SFR altered the fatty acid composition of muscle. In the breast muscle, lower CP levels might favor the deposition of high-nutrient-value unsaturated fatty acids [28]. Compared to CP, SFR had a stronger effect on the fatty acid profile of the muscles. In particular, lower SFR increased SFA content, suggesting that lipid deposition shifted toward a more saturated pattern when dietary fat constituted a larger share of energy supply. This interpretation should also be considered in conjunction with the fatty acid composition of the dietary fat itself, as poultry muscle lipids were strongly influenced by the source and composition of dietary fat [29]. Results from the breast muscle further suggested that an SFR_11:1_ was more conducive to maintaining higher levels of unsaturated fatty acids, while an SFR_5:1_ promoted the accumulation of saturated fatty acids. Therefore, from a meat quality perspective, a medium SFR of 11:1 seemed more ideal.

The experimental diets were formulated to achieve the target starch: fat ratios and protein levels, which inevitably resulted in different inclusion levels of fiber-rich ingredients, including wheat bran, vermiculite (a source of silica and fiber), and rice hull. These fiber sources differ markedly in their chemical composition, particle size, water-holding capacity, and fermentability. As a result, they may influence nutrient digestibility, gut passage rate, and the composition of the cecal microbiota, leading to alterations in short-chain fatty acid (SCFA) production [30,31,32]. SCFAs are known to modulate host energy metabolism, lipid homeostasis, and glucose regulation. Therefore, while our data demonstrate clear associations between SFR and metabolic parameters, we cannot completely exclude the possibility that differences in fiber digestibility and microbial fermentation contributed to the observed effects. Future studies using purified diets or isofibrous formulations would help to isolate the specific role of SFR from that of fiber composition.

Reducing the SFR from 20:1 to 5:1 under low-protein conditions increased subcutaneous fat thickness and the percentage of subcutaneous fat, abdominal fat, and total body fat. Similar increases in fat deposition in response to a lower starch: fat ratio have also been observed in laying hens [33]. Enzymatic results indicated that low-protein diets induced significant changes in liver glucose and lipid metabolism. Compared to a 16.5% crude protein diet, a 14.5% crude protein diet increased AMPK activity but decreased the activities of FAS, CS, and G6PC, suggesting that reduced protein supply caused a certain degree of hepatic energy stress and compensatorily activated AMPK [34]. However, under low protein conditions, the expression of FAS, ACC and PPARα in the liver also decreased with the decrease in SFR, suggesting that the increased fat deposition observed under low SFR was not simply a result of stimulating de novo lipogenesis. Rather, it might reflect an increase in exogenous fat and a redistribution of nutrients to adipose tissue storage [35]. At the same time, the reductions in PPARα and the limited response of CPT-1 suggested that fatty acid oxidation did not increase in parallel with the higher lipid substrate supply. In other words, the oxidative compensation expected under higher fat availability was inadequate, thereby favoring peripheral lipid deposition.

By contrast, under the high-protein condition, the higher PPARα expression observed in the SFR 5:1 group suggests that adequate protein supply may have supported a stronger AMPK-PPARα/CPT-1-mediated oxidative adaptation and thereby improved the capacity for lipid utilization [36]. The apparent inconsistency between ACC activity and ACC mRNA expression might be related to post-translational control, particularly phosphorylation of ACC by AMPK, because enzyme activity was determined not only by transcript abundance but also by protein modification status and the timing of sampling [37,38]. Overall, the present data indicated that low-protein feeding activated liver AMPK-related responses and reduced part of the lipogenic drive, but when SFR was excessively low, the accompanying rise in exogenous lipid supply outpaced oxidative compensation and promoted nutrient partitioning toward adipose deposition. The lack of clear dietary effects on leg fat might be associated with the sequential pattern of fat deposition in poultry, in which fat was preferentially stored in abdominal, intestine, and liver before becoming evident in muscle.

## 5. Conclusions

Optimizing the energy structure of low-protein diets markedly influenced glucose and lipid metabolism in geese. Compared with CP level, SFR more strongly influenced liver cholesterol, LDL-c, glycogen storage, and muscle fatty acid composition. An excessively low SFR (5:1) promoted cholesterol accumulation and body fat deposition, whereas a medium SFR (11:1) was more beneficial for maintaining metabolic balance and a more favorable muscle fatty acid composition.

## Figures and Tables

**Table 1:** Composition and nutrient levels of experimental diets for geese.

Ingredients	Treatments ^1^					
	LPSFR_20:1_	HPSFR_20:1_	LPSFR_11:1_	HPSFR_11:1_	LPSFR_5:1_	HPSFR_5:1_
Corn	50.27	47.25	44.40	41.65	33.10	30.45
Soybean meal	21.70	28.35	21.05	27.50	21.15	26.90
Corn starch	10.50	10.50	10.50	10.50	10.50	10.50
Vermiculite	0.00	1.33	0.00	0.94	2.05	1.88
Wheat bran	6.63	2.40	11.88	8.10	18.00	16.32
Rice hull	6.51	6.37	6.12	5.95	5.75	5.38
Linestone	1.04	0.98	1.05	1.02	1.11	1.07
Dicalcium phosphate	1.34	1.36	1.32	1.31	1.24	1.24
DL-Met	0.16	0.13	0.16	0.14	0.17	0.14
L-Lys	0.16	0.01	0.17	0.02	0.16	0.02
Salt	0.30	0.30	0.30	0.30	0.30	0.30
Soybean oil	0.00	0.00	1.63	1.53	5.00	4.70
L-Leu	0.16	0.00	0.19	0.03	0.24	0.08
L-Thr	0.09	0.00	0.09	0.00	0.09	0.01
L-Try	0.03	0.01	0.03	0.00	0.03	0.00
L-Val	0.11	0.01	0.11	0.01	0.11	0.01
Premix ^2^	1.00	1.00	1.00	1.00	1.00	1.00
Total	100.00	100.00	100.00	100.00	100.00	100.00
Nutrient levels ^3^						
Metabolizable energy (MJ/kg)	10.93	10.91	10.93	10.93	10.96	10.90
Crude protein	14.21	16.34	14.27	16.21	14.40	16.18
Starch	43.14	42.71	41.39	40.59	36.31	33.70
Crude fiber	5.11	5.02	5.10	5.03	5.20	5.20
Lys	0.92	0.92	0.92	0.92	0.92	0.92
Met	0.40	0.40	0.40	0.40	0.40	0.40
Ca	0.84	0.90	0.86	0.91	0.86	0.90
Total Phosphorus	0.66	0.61	0.68	0.63	0.67	0.65
Leu	1.42	1.42	1.42	1.42	1.42	1.42
Thr	0.63	0.63	0.63	0.63	0.63	0.63
Try	0.20	0.20	0.20	0.20	0.20	0.20
Val	0.78	0.78	0.78	0.78	0.78	0.78
Crude fat	2.18	2.12	3.75	3.58	6.78	6.55
Starch: fat ratio	19.79	20.15	11.03	11.34	5.36	5.15

^1^ LPSFR20:1 (CP, 14.5% + SFR, 20:1), HPSFR20:1 (CP, 16.5% + SFR, 20:1), LPSFR11:1 (CP, 14.5% + SFR, 11:1), HPSFR11:1 (CP, 16.5% + SFR, 11:1), LPSFR5:1 (CP, 14.5% + SFR, 5:1), HPSFR5:1 (CP, 16.5% + SFR, 5:1). ^2^ Each kilogram of premix contains VA 1,200,000 IU, VD_3_ 400,000 IU, VE 1 800 IU, VK 150 mg, VB_1_ 60 mg, VB_2_ 600 mg, VB_6_ 200 mg, VB_12_ 1 mg, nicotinic acid 3.0 g, pantothenic acid 900 mg, folic acid 50 mg, choline 35 g, biotin 4 mg, Fe 6 g, Cu 1 g, Mn 9.5 g, Zn 9 g, I 50 mg, Se 30 mg. ^3^ Nutritional levels of crude fat, calcium, starch, apparent metabolizable energy, crude protein, crude fiber, and total phosphorus are measured, and the rest are calculated.

**Table 2:** Primer information for genes.

Gene Name		Primer (5′ → 3′)	Login Number	Length
PPARα	Forward	5′-ATCTATCCCTGGCTTCTCCA-3′	AF481797	117 bp
	Reverse	5′-AGCATCCCATCCTTGTTCATT-3′		
CPT-1	Forward	5′-GTCTCCAAGGCTCCGACAA-3′	GW342945	193 bp
	Reverse	5′-GAAGACCCGAATGAAAGTA-3′		
CS	Forward	5′-TGGTCCCACAACTTCACCAACA-3′	XM_066985720	158 bp
	Reverse	5′-GCGAGGTACGGGTCCGAGA-3′		
SREBP-1	Forward	5′-CGAGTACATCCGCTTCCTGC-3′	EU333990	92 bp
	Reverse	5′-TGAGGGACTTGCTCTTCTGC-3′		
ACC	Forward	5′-TCCAGCAGA ACCGCATTGACAC-3′	XM_037371102.1	187 bp
	Reverse	5′-GTATGAGCAGGCAGGACTTGGC-3′		
FAS	Forward	5′-ATGCTTCAGGAGATGGGTATTG-3′	XM_048050305.1	118 bp
	Reverse	5′-CCATCAGTGTTACTCCCAGCA-3′		
β-actin	Forward	5′-GAAATCGTGCGTGACATCAA-3′	XM_013174886.1	198 bp
	Reverse	5′-GCAGGACTCCATACCCAAGA-3′		

**Table 3:** Analysis of body fat composition under low-protein diets.

Item	Live Body Weight (g)	Skin and Subcutaneous Fat Thickness (mm)	Skin and Subcutaneous Fat Yield (%)	Abdominal Fat Yield (%)	Intestinal Fat Yield (%)	Leg Fat Yield (%)	Body Fat Yield (%)
LPSFR_20:1_	4162.50 ^b^	6.42 ^b^	16.87 ^b^	2.73 ^b^	1.87	1.26	20.00 ^b^
LPSFR_11:1_	4239.58 ^a^	6.33 ^b^	17.43 ^ab^	3.39 ^ab^	2.16	1.16	20.75 ^ab^
LPSFR_5:1_	4279.17 ^a^	7.95 ^a^	20.00 ^a^	4.10 ^a^	2.72	1.42	24.14 ^a^
SEM	14.36	0.217	0.539	0.199	0.178	0.054	0.691
*p* value	0.024	<0.001	0.030	0.009	0.137	0.132	0.022

LPSFR_20:1_ (CP, 14.5% + SFR, 20:1), LPSFR_11:1_ (CP, 14.5% + SFR, 11:1), LPSFR_5:1_ (CP, 14.5% + SFR, 5:1). ^a,b^ means with different superscripts within the same row differ significantly (*p* < 0.05).

**Table 4:** Effect of different dietary treatments on serum and liver lipid metabolites of geese at 63 d.

Item	CP (%)	SFR	Serum				Liver			
			TG	T-CHO	HDL-_C_	LDL-_C_	TG	TCHO	HDL-_C_	LDL-_C_
LPSFR_20:1_	14.5	20:1	1.89	4.56	1.49	3.47	5.87	4.71	2.09	0.60
HPSFR_20:1_	16.5	20:1	1.53	4.92	1.76	3.51	5.94	3.85	1.93	0.97
LPSFR_11:1_	14.5	11:1	1.18	4.62	1.74	3.75	6.64	3.16	1.61	2.18
HPSFR_11:1_	16.5	11:1	1.53	4.73	1.43	3.42	6.33	4.25	1.61	2.58
LPSFR_5:1_	14.5	5:1	1.26	4.55	1.74	3.22	5.86	5.79	2.25	3.38
HPSFR_5:1_	16.5	5:1	1.45	5.03	1.58	3.18	6.40	5.04	2.13	4.35
SEM			0.074	0.138	0.146	0.088	0.151	0.214	0.118	0.227
CP (%)	14.5		1.44	4.58	1.66	3.48	6.14	4.55	1.99	2.05 ^b^
	16.5		1.51	4.89	1.59	3.38	6.23	4.38	1.88	2.64 ^a^
SFR	20:1		1.71	4.74	1.63	3.49	5.91	4.28 ^b^	2.01	0.79 ^c^
	11:1		1.35	4.68	1.58	3.59	6.48	3.70 ^b^	1.61	2.38 ^b^
	5:1		1.35	4.78	1.66	3.20	6.16	5.41 ^a^	2.20	3.87 ^a^
*p*-value	CP		0.634	0.289	0.428	0.539	0.747	0.694	0.691	<0.001
	SFR		0.062	0.950	0.776	0.205	0.314	0.003	0.149	<0.001
	Interaction		0.094	0.863	0.021	0.660	0.545	0.060	0.958	0.065

LPSFR_20:1_ (CP, 14.5% + SFR, 20:1), HPSFR_20:1_ (CP, 16.5% + SFR, 20:1), LPSFR_11:1_ (CP, 14.5% + SFR, 11:1), HPSFR_11:1_ (CP, 16.5% + SFR, 11:1), LPSFR_5:1_ (CP, 14.5% + SFR, 5:1), HPSFR_5:1_ (CP, 16.5% + SFR, 5:1). TG, triglycerides; T-CHO, total cholesterol; HDL-C, high-density lipoprotein cholesterol; LDL-C, low-density lipoprotein cholesterol. ^a,b,c^ means with different superscripts within the same row differ significantly (*p* < 0.05).

**Table 5:** Effect of different dietary treatments on liver and muscle glycogen content of geese at 63 d.

Item	CP (%)	SFR	Liver Glycogen	Breast Muscle Glycogen	Leg Muscle Glycogen
LPSFR_20:1_	14.5	20:1	52.10	2.05	1.74
HPSFR_20:1_	16.5	20:1	56.93	1.85	1.54
LPSFR_11:1_	14.5	11:1	51.34	2.41	1.94
HPSFR_11:1_	16.5	11:1	50.04	2.18	1.46
LPSFR_5:1_	14.5	5:1	49.63	2.17	1.83
HPSFR_5:1_	16.5	5:1	41.12	1.87	1.93
SEM			1.500	0.077	0.094
CP (%)	14.5		50.89	2.24	1.83
	16.5		48.42	1.97	1.66
SFR	20:1		54.52 ^a^	1.93	1.64
	11:1		50.69 ^ab^	2.29	1.70
	5:1		45.38 ^b^	2.00	1.89
*p*-value	CP		0.555	0.118	0.344
	SFR		0.043	0.161	0.587
	Interaction		0.177	0.959	0.512

LPSFR_20:1_ (CP, 14.5% + SFR, 20:1), HPSFR_20:1_ (CP, 16.5% + SFR, 20:1), LPSFR_11:1_ (CP, 14.5% + SFR, 11:1), HPSFR_11:1_ (CP, 16.5% + SFR, 11:1), LPSFR_5:1_ (CP, 14.5% + SFR, 5:1), HPSFR_5:1_ (CP, 16.5% + SFR, 5:1). ^a,b^ means with different superscripts within the same row differ significantly (*p* < 0.05).

**Table 6:** Effect of different dietary treatments on enzymes activity related to liver glucose and lipid metabolism of geese at 63 d.

Item	CP (%)	SFR	FAS (U/mL)	AMPK (U/L)	ACC (U/L)	CS (U/L)	G6PC (U/L)
LPSFR_20:1_	14.5	20:1	1768.64	136.88	38.14	18.41	534.37
HPSFR_20:1_	16.5	20:1	2564.09	81.23	28.68	27.46	899.23
LPSFR_11:1_	14.5	11:1	1653.48	131.33	42.14	19.39	526.49
HPSFR_11:1_	16.5	11:1	2420.00	90.63	29.26	25.31	775.14
LPSFR_5:1_	14.5	5:1	1537.58	123.43	41.28	18.26	505.32
HPSFR_5:1_	16.5	5:1	2280.00	96.29	30.85	25.18	786.40
SEM			88.471	4.996	1.435	0.857	34.968
CP (%)	14.5		1653.23 ^b^	130.55 ^a^	40.52 ^a^	18.68 ^b^	522.06 ^b^
	16.5		2421.44 ^a^	89.38 ^b^	29.60 ^b^	25.99 ^a^	820.26 ^a^
SFR	20:1		2166.36	109.05	33.41	22.94	716.80
	11:1		2001.90	110.98	35.70	22.35	650.81
	5:1		1908.79	109.86	36.06	21.72	645.86
*p*-value	CP		<0.001	<0.001	<0.001	<0.001	<0.001
	SFR		0.232	0.978	0.603	0.731	0.446
	Interaction		0.984	0.309	0.826	0.586	0.626

LPSFR_20:1_ (CP, 14.5% + SFR, 20:1), HPSFR_20:1_ (CP, 16.5% + SFR, 20:1), LPSFR_11:1_ (CP, 14.5% + SFR, 11:1), HPSFR_11:1_ (CP, 16.5% + SFR, 11:1), LPSFR_5:1_ (CP, 14.5% + SFR, 5:1), HPSFR_5:1_ (CP, 16.5% + SFR, 5:1). FAS, fatty acid synthase; AMPK, AMP-activated protein kinase; ACC, acetyl-CoA carboxylase; CS, citrate synthase; G6PC, glucose-6-phosphatase catalytic subunit. ^a,b^ means with different superscripts within the same row differ significantly (*p* < 0.05).

**Table 7:** Effect of different dietary treatments on breast muscle amino acid profile of geese at 63 d. (%).

Item	CP (%)	SFR	Asp	Thr	Ser	Glu	Gly	Ala	Val	Ile	Leu	Tyr	Phe	Lys	His	Arg	Pro
LPSFR20:1	14.5	20:1	1.74	0.87	0.74	2.75	1.05	1.17	0.96	1.11	1.69	0.56	1.11	1.70	0.57	1.34	0.48
HPSFR20:1	16.5	20:1	1.66	0.83	0.72	2.65	1.05	1.13	0.91	1.04	1.61	0.53	1.09	1.66	0.54	1.29	0.46
LPSFR11:1	14.5	11:1	1.69	0.85	0.74	2.65	1.05	1.14	0.89	1.02	1.60	0.52	1.01	1.60	0.52	1.30	0.47
HPSFR11:1	16.5	11:1	1.69	0.86	0.75	2.66	0.99	1.11	0.92	1.03	1.62	0.54	1.08	1.67	0.56	1.27	0.42
LPSFR5:1	14.5	5:1	1.76	0.88	0.76	2.75	1.00	1.14	0.76	1.05	1.65	0.58	1.02	1.64	0.54	1.29	0.41
HPSFR5:1	16.5	5:1	1.67	0.84	0.73	2.61	0.97	1.10	0.90	1.01	1.59	0.54	1.01	1.61	0.51	1.26	0.34
SEM			0.021	0.009	0.008	0.028	0.156	0.011	0.029	0.012	0.017	0.009	0.013	0.016	0.009	0.012	0.014
CP (%)	14.5		1.73	0.87	0.75	2.72	1.04	1.15	0.91	1.06	1.65	0.55	1.04	1.65	0.54	1.31	0.46
	16.5		1.67	0.84	0.73	2.64	1.00	1.12	0.88	1.03	1.61	0.53	1.06	1.65	0.54	1.28	0.41
SFR	20:1		1.70	0.85	0.73	2.70	1.05	1.15	0.94	1.07	1.65	0.54	1.10	1.68	0.55	1.31	0.47 ^b^
	11:1		1.69	0.85	0.74	2.66	1.02	1.12	0.91	1.02	1.61	0.53	1.05	1.64	0.54	1.29	0.45 ^b^
	5:1		1.71	0.86	0.75	2.68	0.99	1.12	0.83	1.03	1.62	0.56	1.01	1.62	0.52	1.28	0.37 ^a^
*p*-value	CP		0.261	0.247	0.269	0.176	0.194	0.244	0.533	0.302	0.345	0.437	0.803	0.846	0.548	0.196	0.048
	SFR		0.242	0.221	0.336	0.164	0.107	0.312	0.201	0.120	0.188	0.063	0.065	0.279	0.322	0.415	0.002
	Interaction		0.711	0.448	0.438	0.577	0.611	0.939	0.459	0.441	0.459	0.413	0.377	0.465	0.357	0.909	0.268

LPSFR_20:1_ (CP, 14.5% + SFR, 20:1), HPSFR_20:1_ (CP, 16.5% + SFR, 20:1), LPSFR_11:1_ (CP, 14.5% + SFR, 11:1), HPSFR_11:1_ (CP, 16.5% + SFR, 11:1), LPSFR_5:1_ (CP, 14.5% + SFR, 5:1), HPSFR_5:1_ (CP, 16.5% + SFR, 5:1). Asp, aspartic acid; Thr, threonine; Ser, serine; Glu, glutamic acid; Gly, glycine; Ala, alanine; Val, valine; Ile, isoleucine; Leu, leucine; Tyr, tyrosine; Phe, phenylalanine; Lys, lysine; His, histidine; Arg, arginine; Pro, proline. ^a,b^ means with different superscripts within the same row differ significantly (*p* < 0.05).

**Table 8:** Effect of different dietary treatments on leg muscle amino acid profile of geese at 63 d. (%).

Item	CP (%)	SFR	Asp	Thr	Ser	Glu	Gly	Ala	Val	Ile	Leu	Tyr	Phe	Lys	His	Arg	Pro
LPSFR_20:1_	14.5	20:1	2.00	1.01	0.87	3.14	1.00	1.25	1.02	1.19	1.87	0.66	1.16	1.86	0.66	1.48	0.49
HPSFR_20:1_	16.5	20:1	1.99	1.00	0.84	3.09	0.98	1.23	1.06	1.17	1.86	0.67	1.21	1.94	0.70	1.49	0.53
LPSFR_11:1_	14.5	11:1	1.97	0.99	0.85	3.08	1.00	1.24	1.05	1.21	1.86	0.66	1.14	1.87	0.67	1.47	0.55
HPSFR_11:1_	16.5	11:1	1.93	0.96	0.82	3.01	0.98	1.22	1.04	1.18	1.83	0.64	1.17	1.86	0.67	1.44	0.55
LPSFR_5:1_	14.5	5:1	1.90	0.96	0.83	2.97	1.00	1.20	1.02	1.17	1.80	0.61	1.12	1.81	0.63	1.41	0.52
HPSFR_5:1_	16.5	5:1	1.95	0.98	0.83	3.06	1.00	1.20	1.04	1.20	1.85	0.64	1.13	1.82	0.67	1.46	0.55
SEM			0.021	0.010	0.009	0.032	0.011	0.010	0.010	0.012	0.017	0.008	0.011	0.017	0.011	0.015	0.006
CP (%)	14.5		1.96	0.99	0.85 ^a^	3.06	1.00	1.23	1.03	1.19	1.85	0.64	1.14	1.84	0.65	1.45	0.52
	16.5		1.96	0.98	0.83 ^b^	3.05	0.99	1.22	1.05	1.18	1.84	0.65	1.17	1.88	0.68	1.46	0.54
SFR	20:1		2.00	1.00	0.85	3.11	1.00	1.24	1.04	1.18	1.86	0.66	1.19	1.90	0.68	1.49	0.51 ^b^
	11:1		1.95	0.98	0.83	3.04	0.99	1.23	1.05	1.19	1.84	0.65	1.16	1.87	0.67	1.45	0.55 ^a^
	5:1		1.92	0.97	0.83	3.01	0.99	1.20	1.04	1.18	1.82	0.63	1.13	1.81	0.68	1.44	0.54 ^a^
*p*-value	CP		0.265	0.119	0.036	0.220	0.345	0.338	0.736	0.739	0.430	0.989	0.161	0.988	0.415	0.430	0.069
	SFR		0.220	0.322	0.774	0.371	0.885	0.244	0.464	0.626	0.316	0.123	0.278	0.101	0.292	0.201	<0.001
	Interaction		0.313	0.338	0.364	0.221	0.803	0.551	0.957	0.784	0.558	0.528	0.986	0.594	0.683	0.424	0.096

LPSFR_20:1_ (CP, 14.5% + SFR, 20:1), HPSFR_20:1_ (CP, 16.5% + SFR, 20:1), LPSFR_11:1_ (CP, 14.5% + SFR, 11:1), HPSFR_11:1_ (CP, 16.5% + SFR, 11:1), LPSFR_5:1_ (CP, 14.5% + SFR, 5:1), HPSFR_5:1_ (CP, 16.5% + SFR, 5:1). Asp, aspartic acid; Thr, threonine; Ser, serine; Glu, glutamic acid; Gly, glycine; Ala, alanine; Val, valine; Ile, isoleucine; Leu, leucine; Tyr, tyrosine; Phe, phenylalanine; Lys, lysine; His, histidine; Arg, arginine; Pro, proline. ^a,b^ means with different superscripts within the same row differ significantly (*p* < 0.05).

**Table 9:** Effect of different dietary treatments on breast muscle fatty acid profile of geese at 63 d (%).

Item	LPSFR_20:1_	HPSFR_20:1_	LPSFR_11:1_	HPSFR_11:1_	LPSFR_5:1_	HPSFR_5:1_	SEM	CP (%)		SFR			*p*-Value		
								14.5	16.5	20:1	11:1	5:1	CP	SFR	Interaction
C4:0	0.182	0.044	0.540	0.287	0.308	0.198	0.036	0.343 ^a^	0.176 ^b^	0.113 ^b^	0.414 ^a^	0.253 ^ab^	0.001	<0.001	0.339
C6:0	0.241	0.204	0.176	0.194	0.238	0.621	0.041	0.218	0.340	0.223 ^ab^	0.185 ^b^	0.429 ^a^	0.054	0.007	0.019
C8:0	1.219	0.484	1.785	1.324	1.069	1.406	0.166	1.358	1.071	0.851	1.555	1.237	0.391	0.238	0.397
C10:0	1.635	1.066	2.977	0.567	1.725	1.644	0.213	2.112 ^a^	1.092 ^b^	1.351	1.772	1.685	0.007	0.562	0.025
C11:0	0.859	1.130	0.526	1.174	0.261	0.667	0.115	0.549	0.99	0.995	0.85	0.464	0.05	0.133	0.763
C12:0	0.151	0.083	0.135	0.299	0.198	0.184	0.021	0.161	0.188	0.117	0.217	0.191	0.464	0.095	0.047
C13:0	0.053	0.035	0.074	0.061	0.051	0.052	0.004	0.059	0.049	0.044 ^b^	0.067 ^a^	0.051 ^ab^	0.144	0.032	0.492
C14:0	0.531	0.374	0.617	0.406	0.512	0.354	0.025	0.553 ^a^	0.378 ^b^	0.452	0.512	0.433	<0.001	0.197	0.779
C14:1 n-5	0.122	0.078	0.153	0.094	0.111	0.096	0.006	0.129 ^a^	0.089 ^b^	0.100 ^b^	0.124 ^a^	0.103 ^ab^	<0.001	0.028	0.05
C15:0	0.172	0.131	0.215	0.153	0.153	0.132	0.009	0.180 ^a^	0.139 ^b^	0.151	0.184	0.142	0.022	0.12	0.597
C15:1 n-5	0.132	0.088	0.156	0.082	0.086	0.065	0.009	0.125 ^a^	0.078 ^b^	0.11	0.119	0.075	0.002	0.12	0.259
C16:0	0.123	0.103	0.179	0.127	0.165	0.134	0.009	0.156 ^a^	0.121 ^b^	0.113	0.153	0.149	0.044	0.099	0.708
C16:1 n-7	0.025	0.017	0.037	0.022	0.025	0.023	0.002	0.029 ^a^	0.021 ^b^	0.021	0.03	0.024	0.012	0.067	0.236
C17:0	0.062	0.046	0.068	0.034	0.036	0.035	0.004	0.055 ^a^	0.038 ^b^	0.054 ^a^	0.051 ^a^	0.036 ^b^	0.009	0.046	0.094
C17:1 n-7	0.064	0.433	0.082	0.049	0.051	0.052	0.003	0.066 ^a^	0.048 ^b^	0.054 ^ab^	0.065 ^a^	0.052 ^b^	<0.001	0.031	0.010
C18:0	0.844	0.594	0.840	0.717	0.842	1.188	0.049	0.842	0.833	0.719 ^b^	0.779 ^ab^	1.015 ^a^	0.901	0.008	0.008
C18:1 n-9t	0.642	0.25	1.023	0.566	0.330	0.285	0.081	0.665 ^a^	0.367 ^b^	0.446 ^ab^	0.795 ^a^	0.308 ^b^	0.037	0.022	0.411
C18:2 n-6t	1.951	0.842	2.829	1.965	1.626	1.816	0.187	2.135	1.541	1.396	2.397	1.721	0.081	0.057	0.241
C18:2 n-6c	2.249	1.891	4.376	2.519	4.060	2.771	0.253	3.562 ^a^	2.394 ^b^	2.070 ^b^	3.448 ^a^	3.416 ^a^	0.006	0.01	0.276
C20:0	0.092	0.232	0.319	0.080	0.065	0.085	0.038	0.159	0.132	0.162	0.200	0.075	0.719	0.376	0.123
C18:3 n-3	0.020	0.018	0.018	0.014	0.014	0.024	0.002	0.018	0.019	0.019	0.016	0.019	0.822	0.848	0.391
C21:0	0.010	0.012	0.017	0.033	0.051	0.048	0.006	0.026	0.031	0.011 ^b^	0.025 ^ab^	0.050 ^a^	0.607	0.018	0.722
C20:2	0.020	0.035	0.041	0.038	0.067	0.038	0.005	0.043	0.037	0.028	0.040	0.053	0.538	0.093	0.165
C20:3 n-6	0.033	0.095	0.064	0.12	0.124	0.119	0.018	0.073	0.111	0.064	0.092	0.121	0.325	0.472	0.722
C22:1 n-9	0.034	0.024	0.148	0.073	0.046	0.038	0.014	0.076	0.045	0.029 ^a^	0.110 ^b^	0.042 ^ab^	0.211	0.028	0.443
C20:3 n-3	0.039	0.087	0.036	0.041	0.063	0.034	0.011	0.046	0.054	0.063	0.039	0.049	0.726	0.679	0.403
C20:4 n-6	0.018	0.074	0.099	0.068	0.026	0.060	0.011	0.048	0.067	0.046	0.083	0.043	0.390	0.270	0.269
C23:0	0.086	0.047	0.036	0.05	0.034	0.022	0.011	0.052	0.040	0.066	0.043	0.028	0.573	0.381	0.628
C22:2 n-6	0.016	0.022	0.023	0.039	0.043	0.038	0.004	0.027	0.033	0.019	0.031	0.041	0.548	0.16	0.609
C24:0	0.100	0.038	0.014	0.040	0.047	0.059	0.008	0.054	0.046	0.069	0.027	0.053	0.571	0.059	0.030
C20:5 n-3	0.033	0.035	0.027	0.046	0.025	0.034	0.005	0.028	0.038	0.034	0.036	0.030	0.373	0.89	0.816
C24:1 n-9	0.036	0.022	0.034	0.045	0.025	0.072	0.006	0.032	0.046	0.029	0.040	0.048	0.200	0.394	0.114
C22:6 n-3	0.070	0.123	0.022	0.024	0.052	0.097	0.019	0.048	0.081	0.097	0.023	0.074	0.417	0.326	0.855
SFA	0.019	0.029	0.037	0.168	0.078	0.083	0.016	0.045	0.093	0.024	0.103	0.081	0.087	0.074	0.125
MUFA	0.380	0.240	0.659	0.78	0.168	0.390	0.101	0.402	0.470	0.310	0.720	0.279	0.746	0.175	0.76
PUFA	1.275	1.968	0.540	0.183	1.772	0.278	0.189	1.196	0.810	1.622 ^a^	0.362 ^b^	1.025 ^ab^	0.163	0.004	0.012

LPSFR_20:1_ (CP, 14.5% + SFR, 20:1), HPSFR_20:1_ (CP, 16.5% + SFR, 20:1), LPSFR_11:1_ (CP, 14.5% + SFR, 11:1), HPSFR_11:1_ (CP, 16.5% + SFR, 11:1), LPSFR_5:1_ (CP, 14.5% + SFR, 5:1), HPSFR_5:1_ (CP, 16.5% + SFR, 5:1). C4:0, butyric acid; C6:0, caproic acid; C8:0, caprylic acid; C10:0, capric acid; C11:0, undecanoic acid; C12:0, lauric acid; C13:0, tridecanoic acid; C14:0, myristic acid; C14:1 n-5, myristoleic acid; C15:0, pentadecanoic acid; C15:1 n-5, pentadecenoic acid; C16:0, palmitic acid; C16:1 n-7, palmitoleic acid; C17:0, margaric acid; C17:1 n-7, heptadecenoic acid; C18:0, stearic acid; C18:1 n-9t, elaidic acid; C18:2 n-6t, linolelaidic acid; C18:2 n-6c, linoleic acid; C20:0, arachidic acid; C18:3 n-3, α-linolenic acid; C21:0, heneicosanoic acid; C20:2, eicosadienoic acid; C20:3 n-6, dihomo-γ-linolenic acid; C22:1 n-9, erucic acid; C20:3 n-3, eicosatrienoic acid; C20:4 n-6, arachidonic acid; C23:0, tricosanoic acid; C22:2 n-6, docosadienoic acid; C24:0, lignoceric acid; C20:5 n-3, eicosapentaenoic acid; C24:1 n-9, nervonic acid; C22:6 n-3, docosahexaenoic acid; SFA, total saturated fatty acids; MUFA, total monounsaturated fatty acids; PUFA, total polyunsaturated fatty acids. ^a,b^ means with different superscripts within the same row differ significantly (*p* < 0.05).

**Table 10:** Effect of different dietary treatments on leg muscle fatty acid profile of geese at 63 d (%).

Item	LPSFR_20:1_	HPSFR_20:1_	LPSFR_11:1_	HPSFR_11:1_	LPSFR_5:1_	HPSFR_5:1_	SEM	CP (%)		SFR			*p*-Value		
								14.5	16.5	20:1	11:1	5:1	CP	SFR	Interaction
C4:0	2.249	1.891	4.376	2.519	4.06	2.771	0.253	3.562 ^a^	2.394 ^b^	2.070 ^b^	3.448 ^a^	3.416 ^a^	0.006	0.01	0.276
C6:0	0.092	0.232	0.319	0.08	0.065	0.085	0.038	0.159	0.132	0.162	0.2	0.075	0.719	0.376	0.123
C8:0	0.02	0.018	0.018	0.014	0.014	0.024	0.002	0.018	0.019	0.019	0.016	0.019	0.822	0.848	0.391
C10:0	0.010	0.012	0.017	0.033	0.051	0.048	0.006	0.026	0.031	0.011 ^b^	0.025 ^ab^	0.050 ^a^	0.607	0.018	0.722
C11:0	0.020	0.035	0.041	0.038	0.067	0.038	0.005	0.043	0.037	0.028	0.04	0.053	0.538	0.093	0.165
C12:0	0.033	0.095	0.064	0.120	0.124	0.119	0.018	0.073	0.111	0.064	0.092	0.121	0.325	0.472	0.722
C13:0	0.034	0.024	0.148	0.073	0.046	0.038	0.014	0.076	0.045	0.029 ^a^	0.110 ^b^	0.042 ^ab^	0.211	0.028	0.443
C14:0	0.039	0.087	0.036	0.041	0.063	0.034	0.011	0.046	0.054	0.063	0.039	0.049	0.726	0.679	0.403
C14:1 n-5	0.018	0.074	0.099	0.068	0.026	0.06	0.011	0.048	0.067	0.046	0.083	0.043	0.390	0.270	0.269
C15:0	0.086	0.047	0.036	0.050	0.034	0.022	0.011	0.052	0.040	0.066	0.043	0.028	0.573	0.381	0.628
C15:1 n-5	0.016	0.022	0.023	0.039	0.043	0.038	0.004	0.027	0.033	0.019	0.031	0.041	0.548	0.160	0.609
C16:0	0.100	0.038	0.014	0.040	0.047	0.059	0.008	0.054	0.046	0.069	0.027	0.053	0.571	0.059	0.03
C16:1 n-7	0.033	0.035	0.027	0.046	0.025	0.034	0.005	0.028	0.038	0.034	0.036	0.030	0.373	0.89	0.816
C17:0	0.036	0.022	0.034	0.045	0.025	0.072	0.006	0.032	0.046	0.029	0.040	0.048	0.200	0.394	0.114
C17:1 n-7	0.070	0.123	0.022	0.024	0.052	0.097	0.019	0.048	0.081	0.097	0.023	0.074	0.417	0.326	0.855
C18:0	0.019	0.029	0.037	0.168	0.078	0.083	0.016	0.045	0.093	0.024	0.103	0.081	0.087	0.074	0.125
C18:1 n-9t	0.380	0.240	0.659	0.780	0.168	0.390	0.101	0.402	0.470	0.310	0.720	0.279	0.746	0.175	0.760
C18:2 n-6t	1.275	1.968	0.540	0.183	1.772	0.278	0.189	1.196	0.810	1.622 ^a^	0.362 ^b^	1.025 ^ab^	0.163	0.004	0.012
C18:2 n-6c	0.261	0.163	0.173	1.150	0.431	0.755	0.134	0.288	0.689	0.212	0.661	0.593	0.129	0.316	0.241
C20:0	0.723	0.459	1.673	2.536	0.288	2.322	0.281	0.895	1.772	0.591	2.105	1.305	0.082	0.057	0.172
C18:3 n-3	0.842	1.449	0.465	0.221	0.167	0.835	0.148	0.491	0.835	1.145	0.343	0.501	0.205	0.051	0.305
C21:0	0.378	1.079	1.462	1.075	2.561	1.181	0.259	1.467	1.112	0.729	1.269	1.871	0.485	0.201	0.259
C20:2	1.693	0.751	0.311	0.247	1.407	0.304	0.146	1.137 ^a^	0.434 ^b^	1.222 ^a^	0.279 ^b^	0.856 ^ab^	0.002	0.003	0.080
C20:3 n-6	0.096	0.301	0.258	0.071	0.093	0.280	0.044	0.149	0.217	0.198	0.164	0.186	0.448	0.950	0.150
C22:1 n-9	0.421	0.378	0.082	1.432	0.342	0.627	0.12	0.282 ^b^	0.812 ^a^	0.4	0.757	0.485	0.009	0.272	0.015
C20:3 n-3	0.298	0.261	0.276	0.079	0.400	0.212	0.035	0.325 ^a^	0.184 ^b^	0.28	0.178	0.306	0.046	0.267	0.548
C20:4 n-6	0.420	0.093	0.110	0.039	0.121	0.072	0.028	0.217 ^a^	0.068 ^b^	0.257 ^a^	0.074 ^b^	0.096 ^b^	<0.001	<0.001	<0.001
C23:0	0.173	0.143	0.152	0.279	0.226	0.218	0.020	0.184	0.213	0.158	0.216	0.222	0.453	0.346	0.221
C22:2 n-6	0.103	0.102	0.137	0.178	0.120	0.105	0.011	0.120	0.128	0.102	0.158	0.112	0.688	0.081	0.514
C24:0	0.113	0.163	0.198	0.268	0.272	0.323	0.021	0.194	0.251	0.138 ^b^	0.233 ^ab^	0.297 ^a^	0.102	0.003	0.963
C20:5 n-3	0.022	0.018	0.036	0.044	0.040	0.048	0.003	0.033	0.037	0.020 ^b^	0.040 ^a^	0.044 ^a^	0.420	0.003	0.569
C24:1 n-9	0.033	0.044	0.045	0.071	0.048	0.044	0.004	0.042	0.053	0.039	0.058	0.046	0.166	0.133	0.317
C22:6 n-3	0.054	0.068	0.067	0.087	0.088	0.086	0.006	0.070	0.080	0.061	0.077	0.087	0.360	0.171	0.699
SFA	0.456	0.511	0.455	0.781	0.708	0.784	0.045	0.540	0.692	0.484 ^b^	0.618 ^ab^	0.746 ^a^	0.060	0.038	0.295
MUFA	0.525	0.536	0.131	1.501	0.419	0.758	0.127	0.358 ^b^	0.932 ^a^	0.530	0.816	0.589	0.012	0.501	0.038
PUFA	2.875	2.846	3.086	3.668	2.477	3.195	0.278	2.812	3.236	2.860	3.377	2.836	0.492	0.716	0.867

LPSFR_20:1_ (CP, 14.5% + SFR, 20:1), HPSFR_20:1_ (CP, 16.5% + SFR, 20:1), LPSFR_11:1_ (CP, 14.5% + SFR, 11:1), HPSFR_11:1_ (CP, 16.5% + SFR, 11:1), LPSFR_5:1_ (CP, 14.5% + SFR, 5:1), HPSFR_5:1_ (CP, 16.5% + SFR, 5:1). C4:0, butyric acid; C6:0, caproic acid; C8:0, caprylic acid; C10:0, capric acid; C11:0, undecanoic acid; C12:0, lauric acid; C13:0, tridecanoic acid; C14:0, myristic acid; C14:1 n-5, myristoleic acid; C15:0, pentadecanoic acid; C15:1 n-5, pentadecenoic acid; C16:0, palmitic acid; C16:1 n-7, palmitoleic acid; C17:0, margaric acid; C17:1 n-7, heptadecenoic acid; C18:0, stearic acid; C18:1 n-9t, elaidic acid; C18:2 n-6t, linolelaidic acid; C18:2 n-6c, linoleic acid; C20:0, arachidic acid; C18:3 n-3, α-linolenic acid; C21:0, heneicosanoic acid; C20:2, eicosadienoic acid; C20:3 n-6, dihomo-γ-linolenic acid; C22:1 n-9, erucic acid; C20:3 n-3, eicosatrienoic acid; C20:4 n-6, arachidonic acid; C23:0, tricosanoic acid; C22:2 n-6, docosadienoic acid; C24:0, lignoceric acid; C20:5 n-3, eicosapentaenoic acid; C24:1 n-9, nervonic acid; C22:6 n-3, docosahexaenoic acid; SFA, total saturated fatty acids; MUFA, total monounsaturated fatty acids; PUFA, total polyunsaturated fatty acids. ^a,b^ means with different superscripts within the same row differ significantly (*p* < 0.05).

## Data Availability

The original contributions presented in this study are included in the article. Further inquiries can be directed to the corresponding author.
